# Eco-Cognitive Computationalism: From Mimetic Minds to Morphology-Based Enhancement of Mimetic Bodies

**DOI:** 10.3390/e20060430

**Published:** 2018-06-04

**Authors:** Lorenzo Magnani

**Affiliations:** Department of Humanities, Philosophy Section and Computational Philosophy Laboratory, University of Pavia, 27100 Pavia, Italy; lmagnani@unipv.it; Tel.: +39-0383-986-283

**Keywords:** computation, information, eco-cognitive computationalism, mimetic minds, organic agents, morphological computing, mimetic bodies, co-evolution, simplexity

## Abstract

Eco-cognitive computationalism sees computation in context, exploiting the ideas developed in those projects that have originated the recent views on embodied, situated, and distributed cognition. Turing’s original intellectual perspective has already clearly depicted the evolutionary emergence in humans of information, meaning, and of the first rudimentary forms of cognition, as the result of a complex interplay and simultaneous coevolution, in time, of the states of brain/mind, body, and external environment. This cognitive process played a fundamental heuristic role in Turing’s invention of the universal logical computing machine. It is by extending this eco-cognitive perspective that we can see that the recent emphasis on the simplification of cognitive and motor tasks generated in organic agents by morphological aspects implies the construction of appropriate “mimetic bodies”, able to render the accompanied computation simpler, according to a general appeal to the “simplexity” of animal embodied cognition. I hope it will become clear that eco-cognitive computationalism does not aim at furnishing a final and stable definition of the concept of computation, such as a textbook or a different epistemological approach could provide: I intend to take into account the historical and dynamical character of the concept, to propose an intellectual framework that depicts how we can understand not only the change of its meaning, but also the “emergence” of new forms of computations.

## 1. Computationalism in an Eco-Cognitive Perspective

What I call *Eco-Cognitive Computationalism* sees computation in context, exploiting the ideas developed in those projects that have originated the recent views on embodied, situated, and distributed cognition: computation is seen as active in physical entities appropriately transformed so that they become what I call *cognitive mediators*, in which data can be encoded and decoded to obtain productive results. This eco-cognitive approach to the concept of computation is strictly related to the tradition of research concerning the so-called distributed and embodied cognitive systems. The “distributed” approach sees cognition as a socially distributed process, pragmatically oriented, and affirms that cognitive processes can be better analyzed as situated in and distributed across material artifactual circumstances, in which the “ecological” view also emphasizes the role of the agent-environment interaction. The theory of distributed cognition was created by Edwin Hutchins [1,2] to describe in a novel and more satisfactory way various problem solving processes in real work situations, so providing a new perspective that encountered great success in general cognitive science. I consider this approach particularly appropriate to treat the concept of computation: I will try to show that using this perspective both Turing’s discoveries and the recent new deal on morphological computation can be better understood and at the same time seen as sharing analogous epistemological characters. Indeed, eco-cognitive computationalism does not reduce computation to digital computation (that is to the processing of strings of digits according to rules), but it is open to include and consider other and new forms of computation. The perspective of the ecology of cognition concentrates on “physical computation”, exactly following Turing’s original thoughts concerning the emergence of computation in organic, inorganic, and artefactual agents, I will quickly describe in this article.

Turing’s germinal speculations on how the so-called “unorganized brains” and “unorganized machines” (On the meaning of these concepts in Turing’s sense, see Section 2.1 and Section 5 below) are transmuted in organized “machineries” are extremely interesting: the problem is to see how, so to speak, “innocent” entities (in the sense of virginal objects “free from cognitive capacities”) become first of all carriers of information and knowledge, but also carriers of computation. Turing is convinced that the emergence of rudimentary forms of information and cognition can be clearly comprehended in an evolutionary perspective, as the fruit of an eco-cognitive interplay and simultaneous *coevolution*, in time, of the states of brain/mind, body, and external environment. At the same time, this evolutionary perspective favors the subsequent creation of both the Universal Logical Computing Machine (which illustrates computation as a pure syntactic process) and the concrete Universal Practical Computing Machine (Further details on this view proposed by Turing are illustrated in [3]). Machines lose their cognitive innocence (they were already merely involved in elementary low level cognitive tasks, such as in the case of telephone, telegraph, etc.) and become *universal* cognitive physical entities, in so far as they become *computational* artefacts that compute for humans or artefactual agents: those computers that in this perspective offered by Turing I called “mimetic minds” [4] (the concept of mimetic mind is explained below in the following Section 2). I hope it will become clear that eco-cognitive computationalism does not aim at furnishing a final and stable definition of the concept of computation, such as a textbook or a different epistemological approach could provide: I intend to take into account the historical and dynamical character of this concept, to propose an intellectual framework that depicts how we can understand not only the change of its meaning, but also the “emergence” of new forms of computations.

## 2. The Birth of *Mimetic Minds*: Educating Human Brains/Educating Physical Entities

I have said in the previous section that what I call *eco-cognitive computationalism* sees computation as active in physical entities suitably transformed so that they become what I call *cognitive mediators*, in which data can be encoded and decoded to obtain useful results. I also stressed the importance of an evolutionary framework that sees the emergence of information, cognition, and computation as the result of a complex eco-cognitive interaction and simultaneous coevolution, in time, of the states of brain/mind, body, and external environment. As I have stressed in [3], the concepts of information, cognition, and computation must not be considered as static, and their meaning changes depending on the modifications of theory and practice: in this article, I will present the new variation of the meaning of the concept of computation generated by the involvement of morphological aspects.

Following the Peircean semiotic perspective, we can remember that signs can be externalized in both natural and artefactual environments and we could add, using a term coming from the last decades of research in cognitive science, that signs are *distributed* and so externalized in a process also called “disembodiment of the mind”. In his seminal article *Intelligent Machinery* (1948) [5], Turing illustrates this same process adopting some neurological metaphors: a big cortex can present an evolutionary advantage when two conditions are fulfilled: (1) the presence of a huge quantity of relevant signs (information and knowledge) available in an environment suitably “artificialized” (props, supports, etc.); and (2) the embedment, discretely developed, in a small community able to manage information (In [4], I have indicated that this speculative argumentation has been recently supported by research in paleoanthropology on the birth of material culture).

The birth of computation is indeed interestingly linked—in Turing’s article I have just quoted—to creative heuristics intertwined with the analysis of the function—out there, in the external environment—of organic bodies, physical entities, and artefacts.

### 2.1. Educating Human Brains

Turing contends that human intelligence is obviously generated by an appropriate education ([5], p. 3) and an analogy between human brains as organic entities and computational machines as physical artefacts has to be built. Brains of the human infant are similar to what he calls *unorganized* machines—machines which are largely random in their construction—that can be educated thanks to “rewards and punishments”. The analogy created by Turing “the cortex of an infant is an unorganized machine” ([5], p. 16) is speculative and just plays a central heuristic role in his creative cognitive processes: obviously, we know from basic neuroscientific research that the brain is a highly organized system. Turing just refers to the fact that the cortices of infants are kinds of blank slates which are “socially” fulfilled through language. From this perspective, the hypothesized random construction of the infant cortex does not deal with neurobiological aspects but with the absence of “education” coming from the external social world—for example, language. Thus, the definition of “unorganized machinery” is related to a special level of description.

When an unorganized machine (such as an infant cortex) is submitted to suitable interferences, its behavior changes and step after step the machine becomes organized (and possibly also universal). Not only is the existence of a human cortex evolutionarily justifiable only in terms of its organization in the framework of a *coevolution* between it and the external information available to organize it: “[…] the possession of a human cortex (say) would be virtually useless if no attempt was made to organize it. Thus if a wolf by a mutation acquired a human cortex there is little reason to believe that he would have any selective advantage” ([5] p. 16). An environment full of information (made real thanks to speech and at the same embedded in a *social background* in which several “techniques” are usable and learnable) has to be already available, but also, as I have noted above, together with a small community that can grant the passage of information across generations.

We have said that, to educate an infant brain, rewards and punishments are needed: this fact indicates that organization can happen only through those two inputs and, finally, the unorganized human cortex is changed in an intelligent one thanks to *discipline* and *initiative*, which Turing considers the two main aspects of a process that has to be studied as it happens in humans to “copy it in machines” ([5], p. 21).

### 2.2. Educating Physical Entities

It is natural for Turing to employ the idea of education in the case of humans to construct the new concept of computation: also physical machines can be *educated* to produce certain kinds of modification. In this last case, education coincides with programming, which is an imitation of the human case: we can modify machines thanks to programming to the aim of reaching some good and interesting responses. The concept of (Universal) Logical Computing Machine (LCM) emerges in this epistemological atmosphere and, on the basis of this abstract theoretical tool, also one of the (Universal) Practical Computing Machines (PCM). These are digital computers (as physical entities) that can be built and “educated”, able to imitate the behavior of a human computer very well. I have already anticipated that, to show Turing’s externalization of both the abstract (the logical machine) and the concrete (the practical machine) entities, I have called them *mimetic minds* [4,6]: these machines are indeed capable of imitating the mind in a universal manner and are objectified in the environment, as clearly expressible formal intellectual structures and practical machines. They are universal because we do not need various machines that do different performances: thanks to suitable programming activities, we can have various performances done. The analogy is completed saying that universal machines are subjected to *paper* interference, when the introduction of new information in the machine modifies its behavior, but also (and here the analogy is no longer active) to *screwdriver* interference, when some parts of the machine are eliminated and and replaced by others, giving rise to completely new machines (by the way, in organic agents, this effect of changing the structure is caused by the evolution) (More details on Turing’s ideas summarized in this section are illustrated in ([6], Chapter 3)).

## 3. Building Computational *Mimetic Bodies* through Morphology-Based Enhancing

It is in the framework I have described in the previous section that we can limpidly see—naturally extending Turing’s perspective—that the recent emphasis on the simplification of cognitive and motor tasks generated in organic agents by morphological aspects implies—once exploited in robotics—the need not only of further computational mimesis “*à la* Turing” of the related performances—when possible—but also the construction of appropriate *mimetic bodies* able to render the accompanied and integrated computation simpler, according to a general appeal to the *simplexity* of animal embodied cognition, which stresses possible complementary relationships between complexity and simplicity [7,8]. To comprehend this process, it is first of all necessary to illustrate various aspects of the so-called physical computation, in which we see exactly at play a further detailed description of that “organization of unorganized entities” introduced by Turing.

### 3.1. A Computer Is a Physical System

In my article [3], I have illustrated some aspects of the relationships between the concepts of information, cognition, and computation, trying to ask the question “Is cognition computation?”. We can give a positive answer, it is, but it is not only computation, so that we cannot identify cognition with computation. Of course, information processing and computation are involved in cognition, and plenty of research has been done to clarify the various roles and types of computation and information processing implicated in cognition, even if these kinds of research, at least from an eco-cognitive perspective, are damned to become old-fashioned. I prefer to affirm, from a perspective informed by a kind of naturalistic epistemology, that, given the fact the concept of computation changes and is subject to meaning variations, the other two concepts, already individuated in themselves by precarious definitions, being associated with the developments of knowledge, technologies, and cultural frameworks, also vary. Recent rich and informed studies offered by Fresco [9] and Piccinini [10,11] fruitfully aim at disambiguating the concept of digital computation in contemporary cognitive science, by illustrating how digital computation is implemented in *physical systems*: these studies do not end up in pancomputationalism (Cfr. also below, Section 4), that is, the view that every physical system is a digital computing one and can be described in computational terms (on the related discussions regarding the various recent views on the relationships between computation and representation in neuroscience (and on computationalism with respect to brain, that is the view that the brain too executes “computations”) cf. a recent special issue of the journal *Minds an Machines* [12]).

These studies mainly aim at philosophical/ontological and definitory results. My concern here is more humble: my eco-cognitive computationalism does not aim at furnishing an ultimate and static definition of the concepts of information, cognition, and computation, such as a textbook or a more “analytic” perspective can provide. Instead, by valuing their historical and dynamical aspects, I will suggest an epistemological perspective that depicts how we can understand the change and extension of their meanings thanks to the example (and the description of the “emergence”) of what I call the *new* computational “domestication” of physical entities, thanks to morphological computing. To this aim, I will adopt the perspective on physical computation offered by ([13], p. 14) that is particularly appropriate to favor my concerns.

1. A *computer* is a physical system with actual constituent parts and its own internal interactions that take it from one physical state to another.
2. Hence, I agree with Horsman et al. who contend that physical computing is “the use of a physical system to predict the outcome of an abstract evolution” ([13], p. 14). Indeed, it is interesting to note that in computations we do not have to deal with a physical system that needs to be described (this is, for example, the case of physics), but, on the contrary, with an abstract object that we want to evolve thanks to the physical system itself. Once we have realized this exploitation of a physical entity, the physical evolution performs a computation, so interpreted by a human or another artificial agent.
3. A computer is usually a *technology* built by using our scientific theories to precision-engineer physical systems to desired specifications (I will illustrate more details on this issue below in Section 3.3).
4. A physical system is computing when a special relationship of abstract mathematical/logical entities to the physical ones at stake is enforced.
5. Beyond conventional and unconventional cases (An interesting alternative view of computation is expressed by the so-called Actor model [14]: computation is conceived as distributed in space where computational machines communicate asynchronously and the whole computation is not in any well-defined state. In response to a message that it receives, an Actor can perform local decisions, generate more Actors, send new messages, and choose how to respond to the next message received. Turing’s Model is a particular case of the Actor Model. In this perspective, the Internet performs unconventional computations and seems controlled by super-recursive algorithms [15]), the notion of *computation*, and its related system property, *information*, has been imported into other fields in an attempt to describe and “explain” such diverse processes as photosynthesis and the conscious mind, and a strand of modern cross-disciplines have given us the claims that “everything is information” or “the universe is a quantumcomputer” or “everything computes” ([13], p. 2). Obviously, many researchers plausibly contend that, defining the universe and everything in it as a computer, the notion of *physical computation* becomes *empty*. I will come back to this issue below in Section 4.

### 3.2. Physical Computing vs. Physics

As I have already anticipated, following the approach proposed by Horsman, Stepney, Wagner and Kendon [13], physical computing can be usefully considered the inversion of mathematical science: *a physical system is used to predict the outcome of an abstract dynamics*, on the contrary, in physics, *an abstract model is used to predict physical dynamics*. Indeed, physics works by representing physical systems abstractly, using abstract theories to predict the result of physical evolution. Let us repeat: if physical computing is the inversion of mathematical science, and uses *a physical system to predict the outcome of an abstract dynamics*, without this predictive element, a physical system is not a computer, in the same way that a group of mathematical equations is a bad physical model if it does not have predictive power. Hence, in physical computation, we want to take an abstract entity, a computation, and represent it physically. It is a process of *representation* that grants a possible “comparison between physical processes and mathematical described computations” ([13], p. 2). On the contrary, in physics, for example, an electron is represented as a wave function, constituting a map between physical and abstract spaces.

In summary, in a computation, “the initial impetus is not a physical system that needs to be described, but rather an abstract object that we wish to evolve. An abstract problem is the reason why a physical computer is used” ([13], p. 10). We therefore start immediately with the problem of a reversed representation.

### 3.3. Computational Physical Systems Usually Are Technological Devices

The construction of what Turing called Universal “Practical” Computing Machines is not at all unrelated to the development of technologies able—in this case—to “domesticate” physical artefacts to behave in a certain way: after all, with the aim of having at our disposal physical computations, we need appropriate physical entities able to perform the task, and this is a fruit of technological advances. It is surely astonishing that humans were able to transport the Turing’s abstract machine, as a Discrete-State Machine (DSM) [16]) to physical machines, creating artificial technological processes that are able to evolve by discrete states, thanks to the role played by silicon electronic devices. We have to add some considerations that can help to understand the meaning of the concept of DSM. Since Turing computer science teaches us that physical artefactual entities are built to process the organization of information and knowledge into little boxes, bits and pixels, which present discrete accuracy (each datum is clearly distinguished and accessible and each measure exact, in contrast to what is occurring for all—classical—physical processes), with no fuzziness and no contingency. In chaotic deterministic systems, a fluctuation/variation below the interval of measurement generates strongly different evolutions for a system. Turing notes in the 1950s that this consequence is theoretically evitable in his Discrete State Machine (DSM): The Turing machine is an abstract (logical, he said) machine, endowed with a Laplacian behavior, as a DSM the machine processes values that are inevitably discrete. The concept of a program and its mathematical characters that are related to its implementation are deterministic exactly in Laplace’s sense: the use of rule (or in the case of Laplacian mechanics, of equations, entails predictability).

When a scientific theory in natural sciences is accepted (that is, when it is appropriately confirmed and relatively exempt of intolerable anomalies or falsifications), explanatorily powerful, and usually endowed with predictive capabilities, it is the prototype of the success of progressive scientific research program, as the philosopher of science Imre Lakatos clearly described [17]. We also perfectly know that these good scientific theories are at the basis of engineering: they are not only tools for discovering new physical systems (and to make predictions regarding their behavior), but also for constructing new artefactual physical systems. Scientific theories are used to engineer physical systems that humans can further “domesticate” to obtain technologies endowed with desired specifications and expected behaviors.

In the case of technologies, we do not have to deal with a theory that models a physical system but something reversed: it is necessary to start from a good physical theory T, able to provide an abstract specification of the physical system that we aim at building that can be called $m_{p^{\prime }}$. The aim of technology is to construct the corresponding physical system, $p^{\prime }$, effectively reversing the modeling relation: “The process of technology to produce this reversal consists of finding a physical system p, the theory T and a specific set of evolutions **H** that will perform the evolution $p\to p^{\prime }$ such that, when $p^{\prime }$ is represented using $R_{T}$, it becomes the desired $m_{p^{\prime }}$ (which is an abstract specification of the physical system that we wish to construct, within the representation of the theory at stake, to the aim of de facto reversing the modeling relation). The physical system p is thus engineered using the process H to produce the desired physical system $p^{\prime }$. An example would be taking a set of steel girders and building a bridge out of them” ([13], p. 9). Of course, finding the ways for reaching the final technological artifact requires a project that is fruit of ingenuity and skill on the part of the various actors at play.

#### New Computational Substrates

We know that present standard substrates for computation are highly engineered silicon entities whose behaviors can be explained by very established and reliable physical theories. Now, we are dealing with the problem of finding new physical substrates able to carry computation, the first requirement is the availability of a reliable physical theory of them, able to guarantee good expected functions and their regularity. Because physical computing is the inversion of mathematical science, as I have indicated in the previous subsection, the physical system is used to *predict* the result of an abstract dynamics (rather than an abstract model predicting physical dynamics). Consequently, knowing the predictive capacities of the physical system is fundamental. Horsman et al. usefully describe an “unfortunate” method for attributing computational capacities to a non-standard system:

A novel computing substrate is proposed (a stone, a soap bubble, a large interacting condensed-matter system, etc.) The physical substrate is “set going” and an evolution occurs. At a certain point, the end of the process is declared and measurements taken. The initial and final states of the system are compared, and then a computation and a representation picked such that if the initial state and final states are represented in such a way, then such a computation would abstractly connect them. The system is then declared to have performed such a computation: the stone has evaluated the gravitational constant, the soap bubble has solved a complex optimization problem and so on.

([13], p. 20)

In summary, the computational description of a physical evolution does not have to be enforced “only” post-hoc: “the challenge then for non-standard computation is to demonstrate that the theory of the device, and the representation of data within it, is known and stable enough to use the physical device to predict the desired abstract computation” ([13], p. 21).

### 3.4. Encoding, Decoding, Computational Entities, Unconventional Substrates

Four basic items are indispensable to illustrate the role of coding and decoding when exploiting physical computational entities and the the status of their relationship with the presence of a “computational agent”.

1. To start a computation, it is first of all necessary to initiate a process of *encoding*—which is an act of *representation*—abstract data and *actual embedding* the related problem in a physical system (indeed, we are assured that our artefactual physical system works because—as illustrated in the previous subsection—we have a good physical theory that can predict how encoded data will work). We have to add that encoding and actual embedding constitute the act of putting the problem into a form—thanks to some appropriate cognitive representation, for example mathematical—that can be carried by an abstracts algorithm, which in turn through refinement and composition will be encoded into the machine (the artefactual physical entity that starts to play the role of a computer), which will be able to manage it. It is through this process that the abstract algorithm becomes a concrete algorithm. In the meantime, abstract algorithms are transformed into equivalent concrete algorithms, exactly as Turing already described, and when the artefactual physical system is “domesticated” as a computer, and we can reliably expect to find the computable results of abstract evolutions, we finally face what Turing called universal practical machine (cf. above Section 2). I have already observed that the act of encoding is subjective and related to a particular human or artefactual agent (indeed, the meaningfulness of encoding and decoding information in physical entities can be, for example, performed by non biological but computational entities, such as an AI program).
2. Thanks to the physical evolution of the system—as I have said, these systems are usually artefactual highly engineered silicon devices with an extremely well developed physical theory in which we have a great deal of confidence— we arrive at the final physical state (which is *decoded* as another abstract state). Consequently, this final abstract state did not evolve abstractly, but concretely, physically. Without the encode and decode steps, there is no computation; there is simply an “ignorant”—so to speak—physical system undergoing evolution: “going about its business”, *only potentially computational* ([13], p. 15) (We can usefully add “This is how we can escape from falling into the trap of `everything is information’ or `the universe is a computer’: a system may potentially be a computer, but without an encode and a decode step it is just a physical system” ([13], p. 15). We have to remember that non standard physical artefactual entities that are used as computing devices and substrates—with the exception of quantum computers—have a theory much less developed and, consequently, the reliability is low).
3. Coding and decoding imply the need of a so-called *computational entity* (it can be organic or not: human, animal, artefactual) that is able to represent—and at the same time to delegate and to recognize *meaning* to—that specific physical system as that specific abstract object.
4. Can living organisms of all sorts be considered physical entities that potentially perform information processing, and that can potentially be exploited to perform their computations for us? As I will describe in the following subsection, the physical RC—Reservoir Computing—approach implies that we can exploit the dynamical properties of various kinds of body parts to carry out relevant computational tasks.

### 3.5. Morphology-Based Enhancement of Mimetic Bodies

Cognitive science has clearly illustrated that intelligent abilities in performing behaviors of natural agents is often explained with reference to their bodily structure (morphology). This morphology can be used for the engineering of intelligent abilities in artificial agents, such as robots. Literature has individuated three main roles played by morphology (see, for example, the rich treatment provided by Müller and Hoffmann [18]):

1. morphology that facilitates control, where no control system, no motors and no sensors are present;
2. morphology that facilitates perception;
3. morphological computation in proper sense, such as “reservoir” computing, in which embodiment and computation are strictly connected.

In the first case, an example is the passive dynamic walker (in which the behavior is occurring by purely mechanical interaction); in the second case, the classical case is illustrated by the Gecko feet, which can in turn be considered as active extensions of the passive walker, thanks to actuators and sensors (the specific ability of the Gecko is the result of its morphology interacting with a particular environment—not primarily that of higher-level central control); also the case of the eye of the fly, which not only concerns movement, but also cognitive abilities related to perception, is another good example; the third case refers to the so-called Reservoir Computing, abstractly proposed by the neural network community, and optimally extended to the role of physical devices that can operate as a reservoir (Physical RC (Reservoir Computing)), in which the body is effectively domesticated for computation. Reservoir computing is not only limited to morphological computations, but, as I have just said, originates from research on neural networks, and contemplates a collection of neurons, as Müller and Hoffmann summarize,

[…] with nonlinear activation functions and with recurrent connections that have a random but bounded strength; this is referred to as a dynamic reservoir. These neurons are randomly connected to input streams, and the dynamics of the input is then spread around and transformed in the reservoir, where it resonates (or “echoes”—hence the term “echo-state networks”) for some time. It turns out that tapping into the reservoir with simple output connections is often sufficient to obtain complex mappings of input stream to output stream that can approximate the input–output behavior of highly complex nonlinear dynamical systems. During training, the weights from the input streams and between the reservoir neurons are left intact; only the output weights—from the reservoir to the output layer-are modified by a learning algorithm (e.g., linear regression). The complexity of the training task has been greatly reduced (as opposed to training all the connections) by exploiting the reservoir to perform a spatiotemporal transformation of the input stream (the temporal aspect of the input sequence has basically been unfolded by the reservoir and can be retrieved directly at any instant). Furthermore, if feedback loops from the output back to the reservoir are introduced and subject to training, the network can be trained to generate desired output streams autonomously.

([18], p. 6)

The amazing novelty is that some physical entities can work as a reservoir as well, giving origin to the so-called Physical RC, which can also be the body of an agent: indeed, biological bodies interacting with their environments can present the needed properties—high dimensionality, nonlinearity and fading memory (More updated details can be found in [18] (pp. 3–7) and [19,20]).

### 3.6. The Birth of Mimetic Bodies: Enhancing Ignorant Bodies through Distributed Computation

To further examine the ways of domesticating—beyond the usual world-wide widespread use of silicon devices—non-stardard entities for computation, we need describe some cognitive and epistemological aspects of the so-called RC (Reservoir Computing). In this case, a classical “digital” computational controller tool (also a robot for example)—that already needs its own “conventional” physical embodiment—is enhanced by the fact that some cognitive “virtues” are outsourced to the morphology of physical entities with the aim of getting the good fruits of an “analog” computation. Where should we draw the line between morphological computation and pure digital computation? Hauser et al. ([20], p. 227) furnish the following explanation of the mechanisms of the RC (Reservoir Computing) (Various real physical systems—certainly not explicitly built for computation—can potentially serve as reservoirs: examples of actual implemented reservoirs such as the well-known case of a soft silicone based octopus arm, employed to emulate desired nonlinear dynamical systems and adopted to carry out computations and to implement a feedback controller, are illustrated in [19,20,21]):

At the core of RC lays the so-called *reservoir*, a randomly initialized high-dimensional, nonlinear dynamical system, which maps the typically low-dimensional input (stream) onto its high-dimensional state space in a nonlinear fashion. In that sense the reservoir takes the role of a kernel (in the machine learning sense, i.e., the nonlinear projection of a low dimensional input into a high-dimensional space […]). In addition, the reservoir, being a dynamical system, has the inherent property to integrate input information over time, which is obviously beneficial for any computation that needs information on the history of its input values. It is important to note that the reservoir is not altered during the learning process. Although it is randomly initialized, its dynamic parameters are fixed afterwards. In order to learn to emulate a desired input output behavior (to be more precise, a desired mapping from input streams to output streams), one has to add a linear output layer, which simply calculates a linearly weighted sum of the signals of the high-dimensional state space of the reservoir. These output weights are the only parameters that are adapted during the learning process.

([20], p. 227)

Implementation of the so called Physical RC involves real physical bodies that are used as reservoirs and as computational sources. Obviously, both the entities involved in classical highly engineered silicon devices and in Physical RC exploitation of bodies do not “know” that they are part of a computational device. They simply conform to the laws of physics and they respond spontaneously. In the case of Physical RC, we have to note “[…] that this also implies that the body does not over—nor under—compensate, since it is a stable physical system. The proposed setup simply adds some linear readouts to the body to complete the computation. The body would react exactly the same, if there were no readouts at all. If this output is used, e.g., in a feedback loop as a control signal for the robot, the behavior of the robot of course should be different if we close the loop by adding the readout” ([20], p. 230). It is important to note that, in the case of morphological computation, a physical computer does not need to be intelligently conceived: it can be naturally (or even computationally) evolved. This means that living organisms or parts of organisms (and their artefactual copies) can potentially execute information processing, and can potentially be exploited to execute their computations for us (On this issue, cf. also ([13], pp. 19–22)).

Hence, the body is so to speak “ignorant” ([20], p. 231), it behaves the same whether we add readouts or not and can be used in various computations on the same input at the same time adding the needed readout. Defining the body “ignorant” of the computations that are carried through it can sound awkward to some ears. However, determining the body as an ignorant term of the computation does not automatically imply that we can find a more knowledgeable part of it—nor it should point in that direction. On the contrary, considering the fact that no part of the system knows that is part of the system itself, I think that is significant to point out the “ignorant” feature of the body, as a fundamental part of the system, not with respect to the other “knowledgeable” parts, but in order to indicate a particular state of ignorance that we can attribute to it, which is not just lack of the permanence (and contamination) of information, but a form of passivity to the computation that happens through it.

Moreover, the classical “physical” computational entities (hardware) as silicon devices or robots endowed with an abstract controller (Hauser et al. also usefully note that “Another important point is that classical robot designs (built out of rigid body parts connected with high torque servos) don’t have any deliberate computation in the physical layer, hence, any computation in the robot is carried out only in the abstract controller layer” ([20], p. 234)) are based on the discreteness of *digital* processing and, from the point of view of their macroscopic features, are stable, unperturbable, and robust with respect to the external influences different from the processes of encoding and decoding (see above Section 3.4). On the contrary morphological computation, in the case of Physical RC, is occurring in the continuous reality (without digital complication) as a clear form of very fast *analog* computation, in which the exploitation of real entities at the same time involves obvious limitations and constraints. We also have to note that to weaken the lack of what we call cognitive/computational plasticity (and—so to speak—of “universality” with respect to the classical Turing machines) of the physical adopted reservoir a kind of *morphosis* (just to make a simple example, moving the body into a new posture) ([20], p. 235) is necessary: the morphology could be modified online to get multiple computational benefits, also taking advantage of the possible effects on the whole system caused by the environment.

In summary, the bodies that are, as I illustrated above, “ignorant”, in the framework of morphological computation become *mimetic bodies*, that is, bodies that are able to mime various cognitive mediating tasks. I call them mimetic exactly in Turing sense: Turing says by “mimicking education, we should hope to modify the machine until it could be relied on to produce definite reactions to certain commands” ([5] p. 14). Similarly, mimicking the morphological features we can get computational results: a standard example is furnished by Nakajima et al. [21,22], who have “strictly” mimicked part of the morphology of an octopus using a model of a soft robotic arm inspired by it. Seeing to the new developments of morphological computation, we can surely conclude that we are facing with new interesting epistemological and technological aspects of what we can call *distributed computation*: we see computation more and more distributed in a wide variety of props, tools, bodies, and devices to the aim of new cognitive results. The promise of morphological computation principles in robot design can originate a new generation of robots with better adaptability and restricted number of required control parameters.

## 4. Pancomputationalism Naturalized

The problems of pancomputationalism is nicely summarized in the following passage by Horsman et al.:

There is currently no accepted answer to this question, and an absence of a worked out formalism within which to determine whether a computation is happening physically gives rise to a great deal of confusion when discussing non-standard forms of computation. We can all agree that a laptop running a Matlab calculation and a server processing search engine queries are physical systems performing computation. However, when we move beyond standard and mass produced technology, the question becomes more difficult to answer. Is a protein performing a compaction computation as it folds? Does a photon (quantum) compute the shortest path through a leaf in photosynthesis? Is the human mind a computer? A dog catching a stick? A stone sitting on the floor? One answer is that they all are – that everything that physically exists is performing computation by virtue of its existence. Unfortunately, by thus defining the universe and everything in it as a computer, the notion of physical computation becomes empty. To state that every physical process is a computation is simply to redefine what is meant by a “physical process”—there is, then, no non-trivial content to the assertion. A statement such as “everything is computation” is either false, or it is trivial; either way, it is not useful in determining properties of physical systems in practice.

([13], p. 2)

The assumption of pancomputationalism clearly states that everything is computational [23]. This view is often defended contending that, when we say that everything is computational, we are just describing something as computational as a way of “interpreting” it, and everything can be interpreted that way. Unfortunately, this defense leads pancomputationalism to conflate computational modeling with computational explanation. Other forms of pancomputationalism contend that the whole universe can be considered computational and seem to also claim that information or computation have a kind of priority with respect to physical materiality ([11], p. 5). Gordana Dodig Crnkovic [15,24,25] proposes a richer info-computational view of the universe (a synthesis of pancomputationalism—naturalist computationalism—with informational structural realism) and defends it by observing the central role of computing in nature (natural computing).

As I have observed in [3], taking advantage of an evolutionary perspective and of the Thom’s concepts of the catastrophe theory [26], when we see the case of an infection as a pregnance (mediated by a virus, that is a material/biological medium) that affects healthy subjects, who are the invested saliences that in turn can re-emit that same contagion as a pregnance into the natural niche (in which, in turn, other media such as air or blood are the transmitters), it seems weird to contend that information (or computation) is at play, in this case against paninformationalism and pancomputationalism. One count is to produce a “model” of that event from an informational point of view or by using a computational program, and another count is to produce a biological knowledge of it (In the following section of this article, I will describe the dangers that can emerge by thinking that mimetic computational modeling “is” immediately, ipso facto, scientific knowledge). However, I think that the positions which defend paninformationalism and pancomputationalism are significative for two reasons. The first reason is related to the consideration of natural human evolution, which presents that widespread activity of semiosis (including all kinds of signs, not only the propositional ones) that has been created by humans since the times of our primitive ancestors. In this way, humans have built those voluminous *cognitive niches*, hugely endowed with informational (and more recently, computational) processes [27,28,29] certainly favors an inclination to envisage some ontological status to information and computation. In addition, I contend that thinking in terms of “distributed computation” (an expression I have introduced above in Section 3.6) helps us see pancomputationalism in a more naturalized way, avoiding ontological or metaphysical considerations.

The second reason is epistemological: in the literature, there is even an all-encompassing notion of information, a kind of paninformationalism, in which physical (or biological) information is extrapolated to every state of a physical (or biological) system that is delineated as an information-carrying state [30,31], which cannot be under-evaluated. Indeed, this perspective has favored many excellent results that physicists (for example) [32,33] (and logicians) have reached, for example providing mathematical frameworks for seeing quantum theory in the perspective of the principles of information processing. My only warning does not concern these results, but the possible abuse of the notions of information (and of computation) in physics and biology, a problem extensively illustrated in detail in [16,34], as I will illustrate in the following section.

## 5. Using Physical Computing to Model Physical and Biological Systems

As I have indicated above (Section 2), Turing argumentations are coherent with the illustration of physical computation as the realization in terms of a physical evolution of an abstract computation I described in Section 3. To quickly recall Turing’s seminal ideas, we can say that he contends that a big cortex constitutes an evolutionary advantage only if it is fertilized by a great quantity of meaningful information and knowledge carried by external supports, props, and tools—interacting with it—that only an already “evolved” collective of humans can have. In summary, *information*, but also *cognitive* contents carried thanks to language, signs, icons, etc. have to be more and more available to promote the useful exploitation of a big cortex. Paleoanthropologists such as Mithen [35,36] would also add that storing in the external environment signs and drawings, and manipulating external entities transforming them in artifacts, is the main process that characterizes not only the birth of the so-called material culture, but also the trigger of a fundamental process of *coevolution* between brains and culture—on this issue cf. also the recent rich and informed book written by Laland [37].

Making an analogy to this evolutionary process, Turing contends that transmuting an external physical artefactual entity (such as an electronic physical object) “paper interference” is needed, when the introduction of new information in the machine modifies its behavior. The Turing lucubrations about the “unorganized” brains, described as kinds of blank slates that are socially fulfilled through language are really modern because, even if very abstract and characterized by a dominant heuristic role, show that phylogenetic mechanisms acting in human cognition are crucial and worth being taken into account, an attitude more powerful with respect to the one of traditional philosophical Western schools.

It is important to note that, in this view offered by Turing, the digital machine (a discrete state machine) is certainly an alphabetic machine: its conditions of possibility resort to human evolution towards alphabetic natural language. Longo [16] contends that this fact is at the origin of that tremendous “discretization of knowledge” that the Turing’s achievements have created. The “continuous” natural language is indeed transmuted by the alphabet in something separated into small atoms, which forge letters. These atoms do not present any kind of meaning that instead comes out thanks to their syntactical aggregation made by skilled human agents able to sensibly combine them. This discreteness, typical of digital machines, is the fundamental aspect that motivates their *imitation* power—they are mimetic machines, *mimetic minds*, as I said—and Turing himself contrasted this simple imitation power to the much stronger epistemological power of the *modeling* capacity of mathematics, when he was thinking about the science of morphogenesis.

At this point, the problem of imitation leads us to consider a further aspect of physical computation: when computers are further used to model physical (or biological systems), are we still dealing with imitation or with a kind of reliable production of knowledge that could occasionally be called “scientific”? Let us see more details concerning this problem. We have indeed said in the previous section that physical computation is a transposition of physical evolution for abstract computation, that is, we can say, following Turing, that we “educate” a physical system to perform a computation. Once this task is performed, we can in turn submit a physical (or biological) dynamic of a system to a computation modeling, that is, we can use computers to simulate the behavior of a physical or a biological system. In the meantime, it is important to note that in this case the computational simulator and the physical or biological system simulated interact at the abstract level and what is simulated is a *model* of the physical or biological system, not the system itself ([13], p. 17). We know that computational modeling of physical or biological processes can be extremely useful as a heuristic tool in actual scientific research, also at the creative level (just to make a simple example, to simulate the behavior—through modeling [38]—of a physical system during an experiment), but we have to note that the study of a physical or a biological evolution can not always take advantage of a computational representation. Hence, what is the epistemological status of computational simulation?

The dichotomy between discreteness and continuity involves a reflection upon the other related dichotomy between imitation (as an effect of the computational representation) and intelligibility (as the fruit of scientific knowledge). I will devote the next paragraphs to better describe this important issue, at least from a general theoretical point of view. As Longo [16] illustrates, the digital machine (a discrete state machine) is first of all an alphabetic machine, made possible thanks to the human evolution to alphabetic natural language (of course, we know it is also based on the so-called logical and formal machine). As I have already noted, this fact explains that powerful “discretization of knowledge” that mainly characterizes the “computational turn”.

This discretization, in the case of the mental representations of “concepts”, generated great consequences. We can guess that an isomorphism is established between the mental processes (where of course “phonemes” play a dominant role), which ensure the stability of a concept, and the physical and material processes that ensure the stability of the actual object represented by the concept. A discretization of knowledge that—long before the computational turn—did not have a marginal role in the recent history of human cognition. The internalization of phonemes [39] has been related to the development of the “enhanced working memory” (EWM) appeared about 30,000 years in hominids and seemed to coevolve with the emergence of a *phonological storage capacity* along with consequent language and other modern reasoning abilities such as planning, problem solving/algorithm manipulation, analogy, modeling, holding inner representations, tool-use, and tool-making, but also cross-modal thinking also needed in social tasks. In particular, an increase in phonological storage could also have aided cross-modal thinking (and so hypothetical cognition) and the social tasks caused by the need of the preservation and defense of collective coalitions: “[…] enhanced phonological storage may have freed language from the laconic and its confinement to present tense and simple imperatives to rapidly-spoken speech and the use of future tense—the linking of past, present, and future, and the use of the subjunctive […]. Although real enemy’s actions might be anticipated, imaginary enemies could be envisioned and other intangible terrors could be given life. Great anxieties could arise with novel vistas (e.g., the meaning of life, thoughts of death, life after death, etc.)” ([39], p. 22).

Notwithstanding the triumph of discretization, in western written natural languages but also in philosophical and logical knowledge, from Democritus to Descartes and from the modern XIX century axiomatics to the computational turn, we are still facing the conundrum represented by the fact that, however, this simple reality of small components is actually very complex, as recent scientific research into the dynamical systems theory, quantum physics, and biology demonstrates. For example, it is difficult to study the cell only by referring to its constituents, and also its “wholeness” is fundamental. The suspect is that, when we use computational devices, which are discrete machines, to produce knowledge about physical and biological systems, some serious expressive limits arise: it is unlikely that these machines can play the role of instruments for directly building scientific intelligibility regarding complex objects/systems such as physical and biological entities (and also human cognitive systems themselves) (We also have to note that, in the last few decades, the notion of digital computation certainly played the role of modeling neural activity and of central features of human cognition, but many neuroscientists (for example [40]) strongly contended that neural networks do not perform digital computation at all: in this last case, cognition is trivially seen as being performed by neural networks but is not computation and the approach we need to use to study cognition has to be related to the theory of dynamical systems).

The deep difference between the idea of scientific knowledge as simple *imitation*, as developed by digital machines and their “modeling capacity”, and the idea of scientific knowledge as generation of *rational intelligibility* is a core problem of the dynamical approach [40]. As I already said above, Turing himself emphasized the difference between the simple *imitation* capacity of machines and the *modeling* power of mathematics: the double pendulum, which is a perfect deterministic machine, only expressed by two equations, is sensible to minor variations, below the threshold of observability: it is a typical chaotic deterministic system, and it is extremely difficult to represent it by a mimetic machine [16]. Longo also notes that this is a system sensitive to initial conditions. It can instead described using the mathematics of deterministic chaos, in which determination does not involve predictability: in this system, a process does not follow the same trajectory even if we reiterate it with the same initial conditions, within the limits of physical measures (ibid.). Furthermore, we can also usefully observe that, in the case of Turing machines that simulate such systems, when we restart after having processes analog simulations, using the same initial data, the same already seen trajectory is performed and, on the contrary, the “real” pendulum behavior is completely different, when we restart the pendulum never performs the same trajectory.

Only “mathematical” models can explain the structure of physical causality of the a system at play, and digital simulation only *resembles* or *imitates* causality, always realizing Laplacian and predictable processes. The explanation of this conundrum resorts to the fact that, at the roots of digital data, there is a *discrete* topology, but physical measurement is always an interval that is optimally represented by continuous mathematics. Even if sometimes the use of mathematical equations in physics does not have a predictive power, this provides a knowledge characterized by a rich *qualitative* epistemological value, able to make intelligible the physical causality of the system [41]. Finally, we have to note that, with respect to physics, in the case of biological organisms, the gap between simulation and intelligibility is even worse because the variability is dominant (In [42] Longo contends that, “in biology, in particular, the introduction of information as a new observable on discrete data types has been promoting a dramatic reorganization of the tools for knowledge” and that some consequences of this effect have been induced in life sciences, with particular emphasis on research on cancer).

In summary, digital simulation—even if epistemological useful at the level of intermediate modeling during the processes of scientific research and discovery, as I have already indicated above in this section—produces an epistemologically distorting result due to its simply mimetic quality.

## 6. Conclusions

In this article, taking advantage of an approach I called “eco-cognitive computationalism”, I have illustrated Turing’s original intellectual perspective that furnished the conceptual framework able to show how, thanks to an imitation of the the evolutionary emergence in humans of information, meaning, and of the first rudimentary forms of cognition, the subsequent invention of the Universal Practical Computing Machine is achieved, that computer that, from the perspective offered by Turing, I call “mimetic mind”. In the second part of the article, I have exploited this framework to illustrate the recent results of morphological computation in which the emphasis on the simplification of cognitive and motor tasks has rendered possible the construction of appropriate “mimetic bodies“ able to render accompanied computations simpler, according to a general appeal to the “simplexity” of animal embodied cognition. I have stressed that, in the case of morphological computation, we can surely conclude that we are facing a new activity of what we can call “distributed computation”: the promise of morphological computation principles in robot design can originate a new generation of robots with better adaptability and restricted number of required control parameters. Finally, I have also dedicated a short discussion to the concepts of paninformationalism and pancomputationalism, showing that the framework of distributed computation helps us see them in a more naturalized and prudent perspective, avoiding ontological or metaphysical considerations. The last section of the article is devoted to illustrating the related problems regarding the epistemological limitations of computation modeling when used to simulate the behavior of a physical or a biological system ([13], p. 17).
